# Pharmacokinetics and bioequivalence of two formulations of mifepristone tablets in healthy Chinese subjects under fasting conditions: a single-center, open, randomized, single-dose, double-period, two-sequence, crossover trial

**DOI:** 10.3389/fphar.2024.1479205

**Published:** 2024-10-16

**Authors:** Yufeng Yan, Xiaoshan Zhu, Ping Dong, Cheng Liu, Lingqing Lu, Liyan Zeng, Guiying Chen, Xianmin Meng, Min Liu

**Affiliations:** ^1^ Department of Pharmacy, Shanghai Public Health Clinical Center, Fudan University, Shanghai, China; ^2^ Wuhan Jiulong Humanwell Pharmaceutical Co., Ltd., Wuhan, China; ^3^ Office of Drug Clinical Trials Institution, Shanghai Public Health Clinical Center, Fudan University, Shanghai, China; ^4^ Department of Clinical Research, Shanghai Public Health Clinical Center, Fudan University, Shanghai, China; ^5^ Wuhan Hongren Biopharmaceutical Inc., Wuhan, China; ^6^ Gynaecology and Obstetrics, Shanghai Public Health Clinical Center, Fudan University, Shanghai, China

**Keywords:** pharmacokinetic, bioequivalence, mifepristone, fasting condition, healthy Chinese subjects

## Abstract

**Objective:**

A bioequivalence (BE) study was performed to evaluate the pharmacokinetics, safety, and bioequivalence of two formulations of mifepristone tablets in healthy Chinese volunteers under fasting conditions.

**Methods:**

A single-center, open, randomized, single-dose, double-period, two-sequence, crossover study in healthy subjects under fasting conditions was performed. The subjects received a single fasting dose of mifepristone (10 mg/tablet) during the first and second periods, followed by a 14-day washout period, during which frequent pharmacokinetic (PK) sampling occurred up to 120 h. The pharmacokinetic parameters of mifepristone were calculated based on the plasma drug concentration–time profile. Primary endpoints were the BE of major pharmacokinetic parameters (AUC_0-t_ and AUC_0-∞_) and the maximum observed serum concentration (C_max_). Secondary endpoints were safety parameters.

**Results:**

Forty subjects (34 male and 6 female subjects) were randomly assigned to treatment, with 39 completing the two-period study. After the single administration of mifepristone tablets (test preparation vs. reference preparation) under fasting conditions, the geometric mean ratios (GMRs) of C_max_, AUC_0-t_, and AUC_0-∞_ were 98.76%, 104.28%, and 104.83%, respectively. The primary metabolites of mifepristone (RU42633 and RU42698),the GMRs of C_max_, AUC_0–t_, AUC_0–∞_ were 102.33% and 100.97%, 103.17% and 103.71%, 104.02% and 103.84%, respectively. Similarly, for another metabolite of mifepristone (RU42698), the GMRs of C_max_, AUC_0-t_, and AUC_0-∞_ were 100.97%, 103.71%, and 103.84%, respectively. All 90% confidence intervals (CIs) for the test/reference AUC ratio and C_max_ ratio were within the acceptable range (80%–125%) for BE, which met the requirements of bioequivalence. No serious adverse events (AEs) occurred, and all AEs were classified as level 1 or 2.

**Conclusion:**

The PK parameters of mifepristone and its metabolites (RU42633 and RU42698) were measured using the (GMRs) of AUC_0-t_, AUC_0-∞_, and C_max_ and were similar between the test and reference drug. The two formulations of mifepristone showed good tolerability and a similar safety profile.

**Clinical Trial Registration:**

chinadrugtrials.org.cn, identifier CTR20182413.

## Introduction

Abortion is a common phenomenon. The annual number of abortions worldwide is approximately 56.3 million, with 25% of pregnancies ending in abortion (Sedgh et al., 2016). Many national and international guidelines emphasize that early medical abortion at home is safe, effective, and preferred by women (Lord, Regan, Kasliwal, Massey and Cameron, 2018). Medical abortion, which includes mifepristone and misoprostol from the World Health Organization (WHO) List of Essential Medicines, plays a crucial role in providing access to safe, effective, and acceptable abortion care.

Mifepristone (11β-(4-dimethylamino) phenyl-17β-hydroxy-17-(1- propynyl) estra-4,9-dien-3-one), also known as RU-486, was the first effective antiprogestin. It is a derivative of the progestin norethindrone, which acts as a competitive progesterone receptor antagonist with both antiprogestin and antiglucocorticoid activity. This activity promotes decidual necrosis to weaken implantation, enhances uterine sensitivity to prostaglandins, and softens the cervix (Cheng, Kelly, Thong, Hume and Baird, 1993; Spitz and Bardin, 1993). Mifepristone was approved by the US Food and Drug Administration (FDA) 20 years ago to induce medication abortion, and in 2012, it was approved to control hyperglycemia secondary to hypercortisolism in patients with endogenous hypercortisolism (Cushing syndrome) (Brown et al., 2020). Mifepristone is also reported to have antiproliferative effects in the ovary (Goyeneche, Caron and Telleria, 2007), endometrium (Moe, Vereide, Orbo, Jaeger and Sager, 2009), cervix (Moe et al., 2009), breast (Moe et al., 2009), prostate cancer (Ritch, Brandhagen, Goyeneche and Telleria, 2019), meningiomas (Touat, Lombardi, Farina, Kalamarides and Sanson, 2014), glioblastoma (Check, Wilson, Cohen and Sarumi, 2014; Llaguno-Munive et al., 2020), and psychotic depression (Flores, Kenna, Keller, Solvason and Schatzberg, 2006).

Mifepristone pharmacokinetics were nonlinear, dose-dependent in humans, and appear to be capacity-limited, characterized by rapid absorption and a long half-life of 20–40 h; the efficacy of mifepristone may be influenced by individual differences in pharmacokinetics (Heikinheimo and Kekkonen, 1993; Heikinheimo, Kekkonen and Lahteenmaki, 2003; Holtyn and Weerts, 2019). Until today, there has been a lot of research focusing on enhancing the solubility and oral bioavailability of mifepristone. The polymorph influences its oral bioavailability; poor solubility and oral bioavailability have some undesirable consequences (Xu et al., 2020). Mifepristone-induced effects include cortisol withdrawal symptoms (fatigue, nausea, vomiting, headache, and arthralgia), anti-progesterone effects (endometrial thickening and vaginal bleeding), and changes in thyroid function (Fleseriu et al., 2012). Research has shown that any bioequivalence trial of highly variable drugs is difficult to conduct (Midha, Rawson and Hubbard, 2005).

This study was conducted to establish the bioequivalence of mifepristone tablets (10 mg) developed and produced by Hubei Gedian Renfu Pharmaceutical Co., Ltd. against the mifepristone tablets (10 mg) produced by Resources Zizhu Pharmaceutical Co., Ltd., which were selected as reference preparations by the National Medical Products Administration of China under fasting conditions; although the two preparations contain the same active ingredient, they differ in their manufacturing processes, and this probably affects the rate and extent of the absorption of the active drug. Thus, a bioequivalence trial of these formulations would be necessary to compare the pharmacokinetic behavior and evaluate the bioequivalence and safety of the two preparations after a single oral administration in healthy Chinese subjects.

## Materials and methods

This clinical trial was conducted between January 9 and 11 March 2019 at the Phase I Clinical Trial Center, Shanghai Public Health Clinical Center affiliated with Fudan University, Shanghai, China. The clinical trial was managed and monitored by a professional contract research organization company and strictly implemented the study protocol without any amendments. The participants voluntarily provided written informed consent to participate in this clinical trial, received anonymous random numbers, and retained the right to withdraw their consent at any time without giving reasons. During the analytical phase of the study, the researchers maintained the samples in a blinded manner. Bias was mitigated through the implementation of independent data analysis, precise measurement techniques, and additional rigorous methodological controls. All research data should be retained by the investigator for 5 years after the end of this clinical trial or 2 years after the drug’s market release, including original records of the subjects’ hospitalization, informed consents, case report forms, and drug scores. Unless required by the National Medical Products Administration (NMPA), the researchers shall not damage the documents, transfer the location, or provide data to any party in any form without the written consent of the sponsor.

### Study design

The clinical trial in healthy Chinese fasting volunteers was a single-center, open, randomized, single-dose, double-period, two-sequence, crossover study. Volunteers were screened between 14 and 2 days before dosing, and eligible subjects were admitted to the Phase I intensive care unit 2 days (day-2) before each medication period. Selected participants were randomly divided into two groups before dosing (day-1) and fasted for over 10 h before medication administration. The subjects received a single fasting dose of mifepristone (10 mg/tablet) during the first and second periods, followed by a 14-day washout period, during which frequent pharmacokinetic (PK) sampling occurred up to 120 h. Blood samples were collected using a vacuum-based blood collector with EDTA-K_2_ within 1 h before drug administration and at 21 time points after drug administration (10, 20, 30, and 45 min and 1.0, 1.33, 1.67, 2.0, 2.5, 3.0, 3.5, 4.0, 5.0, 6.0, 8.0, 12.0, 24.0, 48.0, 72.0, 96.0, and 120.0 h), for a total of 22 blood samples per period. Vital signs were assessed at 1 h before dosing and at 8 time points post-dose (2.0 ± 0.5, 4.0 ± 0.5, 12.0 ± 0.5, 24.0 ± 1.0, 48.0 ± 1.0, 72.0 ± 1.0, 96.0 ± 1.0, and 120.0 ± 1.0 h). Subjects were discharged after a safety assessment on the day blood samples were collected at 120 h post-dose in the first period. On the day of blood sample collection at 120 h post-dosing in the second period, participants were required to undergo a physical examination, vital sign assessment, 12-lead electrocardiogram, and relevant laboratory tests. A telephone follow-up is conducted on D14 ± 2 days after the participant’s last dose (including those who withdraw early) to inquire about any subsequent adverse events (AEs). If an AE occurs, it should be recorded and followed up. If an AE occurs during the trial, follow-up should continue until it resolves, stabilizes, or the participant is lost to follow-up.

### Study subjects

The estimation of the sample size was based on a previous study, in which intra-subject variabilities (CVs) of AUC_0-inf_ and C_max_ were calculated to be approximately 26% and 23%, respectively. The estimated maximum CV of mifepristone metabolites was 24%, θ value was 0.95, α value was 0.05, 1-β value was 90%, and the calculated sample size was 35. To account for a 15% drop-out rate, five subjects were added; thus, 40 subjects were randomly assigned to the group.

Subjects were Chinese male and postmenopausal female volunteers aged between 18 and 65 years (inclusive), with a body mass index (BMI) ranging from 18 to 26 kg/m^2^ (inclusive). Postmenopausal female subjects should meet the following conditions: 12 months of natural menopause or 6 months of natural menopause with plasma follicle-promoting hormone level >40 mIU/mL or at least 6 weeks after bilateral oophorectomy with or without hysterectomy. Subjects needed to be in good health subjects, with no history of chronic or serious diseases, as determined by medical history, vital signs, physical examination, laboratory tests (blood biochemistry, urinalysis, hematology, virological screening, alcohol breath test, and drug abuse screening), pregnancy test (only female), 12-lead electrocardiography (ECG), chest X-ray examination, and gynecologic ultrasound examination [B-ultrasound (uterine + bilateral appendage) + transvaginal ultrasonography]. All subjects have no pregnancy plan, must voluntarily take effective contraceptive measures, and should not plan for sperm or ovum donation during the study and for 6 months after the study. Subjects were not allowed to take any medications or supplements throughout the study.

Subjects were ineligible for trial entry if they had a clinically relevant allergy, skin disease, arthritis, lactose intolerance, dysphagia, or gastrointestinal disease that affects drug absorption; known or suspected hypersensitivity to two or more drugs/foods (in particular to any components of mifepristone); a history of substance abuse within the previous 6 months before screening; had undergone surgery, donated blood or comparable blood loss ≥200 mL, received blood transfusion or used blood products; used drugs, smoked tobacco >5 cigarettes, consumed excessive amounts of tea/coffee and/or caffeinated beverages (more than 8 cups, with 1 cup = 250 mL) per day; drank more than 14 units of alcohol per week (1 unit alcohol ≈ 360 mL beer or 150 mL wine or 45 mL 40% spirit) within the previous 3 months before screening; received a vaccine in the preceding 4 weeks before screening; used any prescription/over-the-counter/herbal/vitamins/non-steroidal anti-inflammatory drugs (aspirin, acetaminophen, indomethacin, diclofenac, ibuprofen, mesalazine, celecoxib, naproxen, etc.) in the previous 2 weeks before screening; consumed excessive amounts food or beverages prepared, including pitaya, mango, grapefruit, lime, and carambola within 7 days prior to screening; consumed chocolate or any food or beverages containing caffeine and xanthine during the period of admission from screening to 2 days; used any drugs that inhibit or induce liver CYP3A4 metabolism within 28 days prior to taking the trial medication; followed a special diet that affects drug absorption, distribution, metabolism, and excretion; and were deemed unsuitable for participation by other researchers.

### Study drugs

The test tablet was mifepristone (10 mg Lot No. 20180805, expiration date July 2021), produced by Hubei Gedian Renfu Pharmaceutical Co., Ltd. The reference tablet was mifepristone (10 mg, Lot No. 43161101, expiration date October 2019), a marketed product issued as the reference listed drug (RLD) by the China National Medical Products Administration, manufactured by Resources Zizhu Pharmaceutical Co., Ltd., China.

### Sample analyses

Serial whole blood samples (4 mL each) for the determination of plasma concentrations of mifepristone were collected by venous puncture or an indwelling venous catheter into anticoagulant vacuum vessels.

During the period, whole blood samples were obtained according to the time sequence outlined in the study design. The collected whole blood samples were centrifuged to separate the plasma under conditions of 2°C–8°C (1,700 g, centrifugation for 10 min). The separated upper layer of plasma was then transferred into two cryogenic vials (each containing more than 0.6 mL but not exceeding 1.0 mL; one vial is designated for content measurement, while the other vial serves as a backup). Blood samples should be placed in the centrifuge within 1 h of collection and stored in a −80°C (ranging from −90°C to −60°C) freezer within 1 h post-centrifugation. Once all samples for both periods have been collected, the plasma samples were promptly transported to the analytical testing center for pharmacokinetic analysis.

### Analysis condition

The main liver oxidative metabolites of mifepristone are N-mono-demethylated mifepristone (RU42633) and the 17-propyl side-chain terminal hydroxylated compound (RU42698). The concentrations of mifepristone, RU42633, and RU42698 in plasma were measured, with mifepristone, RU42633 and RU42698 serving as substances for bioequivalence evaluation. Plasma concentrations of mifepristone and its metabolites (RU42633 and RU42698) were analyzed using a validated high-performance liquid chromatography–tandem mass spectrometry (HPLC–MS/MS) method. The linear range was 4.0–2,000 ng/mL, 4.00–1,000 ng/mL, and 1.00–250 ng/mL for mifepristone, RU42633, and RU42698, respectively. The lower limit of quantification was 4.0 ng/mL, 4.0 ng/mL, and 1.0 ng/mL for mifepristone, RU42633, and RU42698, respectively.

Mobile phase A was 0.01% formic acid in water, and mobile phase B was methyl cyanide. The flow rate was set at 1.00 mL/min, and the elution gradient was programmed as follows: 20% mobile phase B for 0.01 min; 60% mobile phase B for 1.4 min; 90% mobile phase B for 3.6 min; and 20% mobile phase B for 2.2 min. The automatic sampler was cleaned with 500 μL methyl alcohol, followed by a 1.0-s soak and 1.0-s rinse before and after aspiration. An ultimate XB-C18 (50 mm × 2.1 mm, 5.0 μm) analytical column was used to achieve favorable chromatographic separation. The temperature of the column was set at 35°C and the autosampler was set at 4°C; the injection volume was 10.0 μL.

The quantitation of mifepristone, RU42633, and RU42698 was achieved by multiple reaction monitoring (MRM) in the positive ion mode with an electrospray ionization source. The optimized parameters were as follows: ion spray voltage of 5,500 V, nebulizing gas at 50 psi; auxiliary gas at 60 psi, curtain gas at 30 psi, collision gas at 9 psi, and a source temperature of 600°C. Quantitation was performed by monitoring the transitions at m/z 430.1–372.1 for mifepristone, m/z 416.1–358.0 for RU42633, and m/z 446.1–388.1 for RU42698 with the de-cluster voltage of 60 v, 90 v, and 100 v and collision energy of 21 eV, 20 eV, and 30 eV, respectively.

### Pharmacokinetic and bioequivalence analysis

PK parameters such as the kinetic parameter peak concentration (C_max_), the area under the concentration–time curve from time zero to the last measurable concentration (AUC_0-t_), the area under the concentration–time curve to infinity (AUC_0-∞_), elimination half-life (T_1/2_), and time to achieve C_max_ (T_max_) were determined. The arithmetic mean, standard deviation, coefficient of variation, median, maximum, minimum, and geometric mean of each parameter were also calculated. The pharmacokinetic parameters of mifepristone were calculated using Phoenix WinNonlin 7.0 with a non-vatrial model. The experimental results were mainly analyzed using descriptive statistics using SAS (SAS Institute, version 9.4). The measurement data will be presented using the number of cases, mean, standard deviation, median, minimum, and maximum values. Categorical data will be described by frequency and constituent ratios.

The main pharmacokinetic parameters of mifepristone, RU42633, and RU42698 after logarithmic transformation were analyzed. The level of significance was set at *p* < 0.05. The 90% confidence intervals (CIs) for the geometric mean ratios (GMRs) of C_max_, AUC_0-t_, and AUC_0-∞_ between the two formulations were obtained and then converted to the ratio scale by antilog transformation. If the 90% CI of the GMRs were completely within the range of 80.00%–125.00%, bioequivalence was established.

## Results

### Study population

**FIGURE 1:**
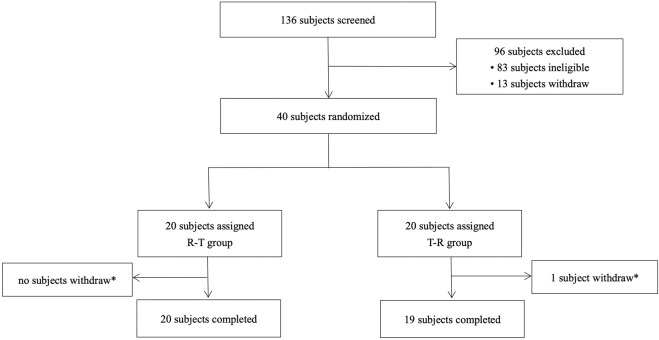
Study subject disposition flow diagram. R, reference drug; T, test drug; *, a subject withdrew due to an adverse event (fever) after the second cycle of administration (24 h after the second cycle of blood sample collection).

A total of 136 subjects were enrolled in this study, with 96 classified as screening failures. The reasons for exclusion are as follows: 83 did not meet the inclusion and exclusion criteria, 5 were excluded due to the screening number being lower, 5 voluntarily withdrew, and 3 were not selected for other reasons. Forty subjects (34 male and 6 female subjects) were randomly assigned to treatment, with 39 completing the two-period study. One subject withdrew due to an adverse event of fever after the second period of administration (Figure 1). Baseline demographics were comparable across the two groups (Table 1).

**TABLE 1:** Demographic characteristics at baseline.

Parameter	Group		Total
	R-T (n = 20)	T-R (n = 20)	
Gender, n (%)			
Male	17 (85%)	17 (85%)	34 (85%)
Female	3 (15%)	3 (15%)	6 (15%)
Chinese, n (%)	20 (100)	20 (100)	40 (100)
Age, years			
Mean (SD)	32.45 (8.46)	33.55 (11.55)	33.0 (10.0)
Min–max	22–50	19–60	19–60
Weight, kg			
Mean (SD)	64.03 (8.98)	63.78 (7.26)	63.90 (8.06)
Min–max	46.4–76.8	50.6–77.7	46.4–77.7
Height, cm			
Mean (SD)	168.15 (8.32)	166.00 (7.15)	167.0 (7.74)
Min–max	152.5–182.0	148.0–178.0	148.0–182.0
BMI, kg/m^2^			
Mean (SD)	22.58 (2.12)	23.13 (1.96)	22.8 (2.03)
Min–max	19.2–25.9	19.5–25.7	19.2–25.9

R, reference drug; T, rest drug; SD, standard deviation; BMI, body mass index.

### Pharmacokinetics calculations

**FIGURE 2:**
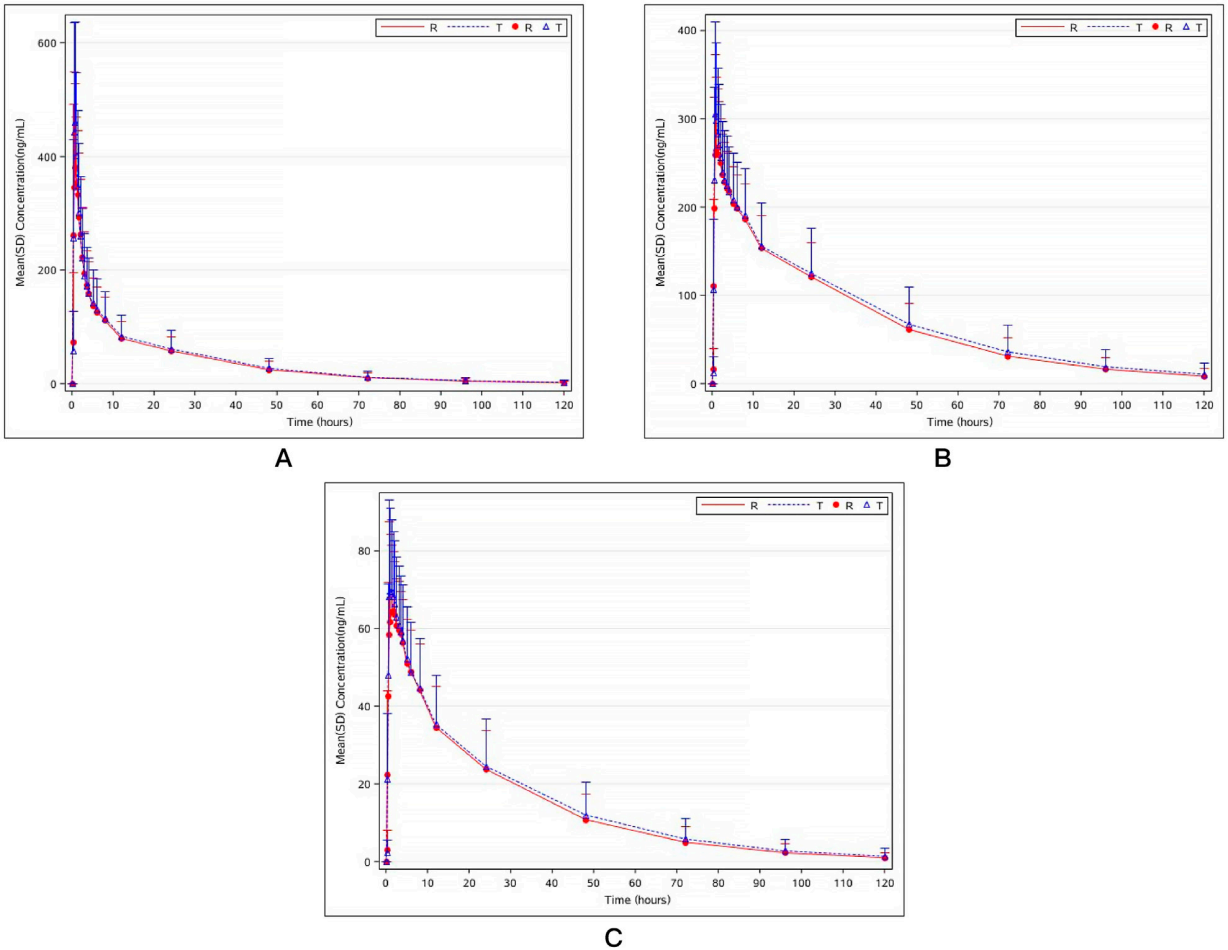
Mean plasma concentration–time curves of mifepristone **(A)**, mono-demethylated mifepristone (RU42633) **(B)**, and hydroxyled metabolite (RU42698) **(C)** after the single fasting oral administration of mifepristone tablet test preparation and reference preparation (10mg).

Two mifepristone tablets, each with a single dose of 10 mg, were administered under fasting conditions. The PK parameters of mifepristone and its metabolites (RU42633 and RU42698) were measured using the GMRs of AUC_0-t_, AUC_0-∞_, and C_max_, which were similar between the test and reference drug. One subject completed blood sample collection in the first period but withdrew 24 h later due to adverse events (fever) in the second period. Only C_max_ was included Pharmacokinetic Analysis Set (PKPS) and Bioequivalence Analysis Set (BES) in the second period (Table 2). Mifepristone was absorbed by the gastrointestinal tract, with the median T_max_ occurring at 0.75 h post-dose under fasting conditions (Figure 2A). The median T_max_ value of the primary metabolite RU42633 occurred at 1.00 h (Figure 2B), while RU42698 was detected earlier at 0.75 h (Figure 2C).

**TABLE 2:** Pharmacokinetic parameters under fasting conditions.

Parameter	Mifepristone, mean ± SD (CV%)		RU42633, mean ± SD (CV%)		RU42698, mean ± SD (CV%)	
	Test (n = 40)	Reference	Test (n = 40)	Reference	Test (n = 40)	Reference
T_max_ (h)	0.75 (0.33–6)	0.75 (0.33–2) (n = 40)	1.00 (0.5–24)	0.75 (0.5–4) (n = 40)	1.00 (0.5–24)	1.00 (0.5–24) (n = 40)
C_max_ (ng/mL)	514.05 ± 177.65 (34.56)	502.68 ± 144.29 (28.70) (n = 40)	335.28 ± 88.08 (26.27)	322.73 ± 65.94 (20.43) (n = 40)	76.42 ± 19.47 (25.48)	74.63 ± 16.58 (22.22) (n = 40)
AUC_0-t_ (h ng/mL)	4538.27 ± 2245.97 (49.49)	4271.00 ± 1821.15 (42.64) (n = 39)	8613.78 ± 3855.26 (44.76)	8148.74 ± 2712.73 (33.29) (n = 39)	1736.80 ± 826.45 (47.58)	1638.69 ± 668.85 (40.82) (n = 39)
AUC_0-∞_ (h ng/mL)	4772.99 ± 2330.84 (48.83)	4473.03 ± 1868.47 (41.77) (n = 39)	9197.47 ± 4415.11 (48.00)	8582.06 ± 3036.85 (35.39) (n = 39)	1814.46 ± 897.32 (49.45)	1702.14 ± 701.80 (41.23) (n = 39)
λ_z_ (h^-1^)	0.0390 ± 0.0131 (33.5711)	0.0399 ± 0.0123 (30.9364) (n = 39)	0.0331 ± 0.0113 (34.0417)	0.0334 ± 0.0112 (33.6973) (n = 39)	0.0331 ± 0.0113 (34.0417)	0.0334 ± 0.0112 (33.6973) (n = 39)
t_1/2z_ (h)	19.80 ± 6.76 (34.14)	19.00 ± 5.82 (30.65) (n = 39)	23.61 ± 8.62 (36.52)	23.14 ± 7.72 (33.37) (n = 39)	20.08 ± 6.84 (34.05)	19.30 ± 6.14 (31.79) (n = 39)

RU42633, mono-demethylated mifepristone; RU42698, hydroxyled metabolite; T_max_, time to C_max;_ T_max_ was represented as the median (min and max) (CV%); C_max_, maximal plasma concentration; AUC_0-t_, area under the concentration–time curve from 0 to t; AUC_0–∞_, area under the concentration–time curve from 0 to infinity; t_1/2z_, terminal elimination half-life.

#### Bioequivalence

All 90% confidence intervals for the GMRs of C_max_, AUC_0-t_, and AUC_0-∞_ of mifepristone, RU42633, and RU42698 were within the range of BE from the FDA guidelines (80%–125%), which met the requirements of bioequivalence (Table 3).

**TABLE 3:** Bioequivalence under fasting conditions.

PK parameter	Mifepristone					Mono-demethylated mifepristone (RU42633)					Hydroxyled metabolite (RU42698)				
	GM and GMR (N = 40)			IIV (%)	90% CI	GM and GMR (N = 40)			IIV (%)	90% CI	GM and GMR (N = 40)			IIV (%)	90% CI
	T	R	GMR (%)			T	R	GMR (%)			T	R	GMR (%)		
C_max_ (ng/mL)	479.23	485.24	98.76	33.30	87.40–111.61	323.04	315.69	102.33	22.32	94.17–111.20	73.72	73.01	100.97	20.78	93.44–109.11
AUC_0-t_ (h ng/mL)	4040.25	3874.25	104.28	24.02	95.28–114.14	7864.06	7622.63	103.17	17.60	96.51–110.28	1555.55	1499.90	103.71	21.47	95.64–112.46
AUC_0-∞_ (h ng/mL)	4276.92	4079.92	104.83	22.17	96.42–113.96	8300.29	7979.74	104.02	17.99	97.17–111.35	1616.63	1556.79	103.84	21.30	95.82–112.54

R, reference drug; T, test drug; GMR, geometric mean ratio of the test over reference pharmacokinetic metric; IIV, intra-individual variation.

#### Safety

Treatment-emergent AEs are presented in Table 4. Before administration, 1 adverse event occurred in one subject (numbered E032). During the trial, 21 adverse events occurred in 9 (22.50%) of the 40 subjects. In the T group, 13 AEs occurred in 6 subjects (15.00%), of which 12 resolved and 1 was of unknown outcome. In the R group, 8 AEs occurred in 3 subjects (7.50%), with 6 cases classified as recovered and 2 as unknown. Four adverse events, reported in two subjects (5.00%, 2/40), were considered important adverse events. One case leading to withdrawal occurred in 1 subject in the R group, and no serious adverse events occurred in both groups. In terms of the severity of AEs, 1 case was classified as level 2 and others as level 1. Among the AEs, four were assessed as possibly related to the medication. Of these, only one was effectively managed through treatment with enzyme-lowering agents due to elevated alanine aminotransferase levels, while the remaining adverse events resolved without the need for intervention.

**TABLE 4:** Summary of treatment-emergent adverse events.

Parameter	N (%) [number of AEs]		
	Test	Reference	Total
Any TEAEs	6 (15.00%) 13	3 (7.5%) 8	9 (22.5) 21
Serious TEAEs	0	0	0
Discontinuations due to TEAEs	0	1 (2.5%) 1	1 (2.5%) 1
Drug-related TEAEs			
Severe TEAEs	0	0	0
All deaths	0	0	0
Positive urine leukocyte	2 (5%) 2	1 (2.5%) 1	3 (7.5%) 3
Urine leukocyte quantification increased	2 (5%) 2	1 (2.5%) 1	3 (7.5%) 3
Elevated urinary bacteria	0	1 (2.5%) 1	1 (2.5%) 1
Urinary occult blood test positive	1 (2.5%) 1	0	1 (2.5%) 1
Fever	0	1 (2.5%) 1	1 (2.5%) 1
Hyperglycemia	0	1 (2.5%) 1	1 (2.5%) 1
Elevated white blood cell count	1 (2.5%) 1	0	1 (2.5%) 1
Low white blood cell count	1 (2.5%) 1	0	1 (2.5%) 1
Absolute number of neutrophils increased	1 (2.5%) 1	0	1 (2.5%) 1
Absolute number of neutrophils decreased	1 (2.5%) 1	0	1 (2.5%) 1
Absolute number of monocytes increased	1 (2.5%) 1	0	1 (2.5%) 1
Elevated alanine aminotransferase levels	0	1 (2.5%) 1	1 (2.5%) 1
Elevated R-glutamylase	0	1 (2.5%) 1	1 (2.5%) 1
Elevated cholinesterase	0	1 (2.5%) 1	1 (2.5%) 1
Elevated creatine kinase	1 (2.5%) 1	0	1 (2.5%) 1
Elevated uric acid	1 (2.5%) 1	0	1 (2.5%) 1
Emesis	1 (2.5%) 1	0	1 (2.5%) 1

## Discussion

Mifepristone tablets, available in 10 mg and 25 mg dosages, are specifically tailored to China’s specifications. Previous studies have confirmed that the test and reference 25 mg mifepristone tablets, produced by the same manufacturers, are bioequivalent, safe, and well-tolerated under fasting conditions in healthy Chinese subjects. The present study aims to further evaluate the bioequivalence of 10 mg mifepristone tablets when administered as a single dose under fasting conditions in healthy Chinese subjects.

According to the recommendations of the Draft Guidance on Bioequivalence of Mifepristone in the guidance on bioequivalence of specific drugs issued by the China National Institute for Food and Drug Control (NFDA), the study should only include healthy men and/or women in menopause because of the anti-pregnancy effect of mifepristone. All the guidelines of the BE study do not contain specific requirements for the gender ratio; hence, the gender ratio was not balanced in this trial. It is worth noting that Chen et al. (2018) found sex-related differences in the pharmacokinetic properties of mifepristone in animals. The ratio of male and female subjects in the R and T groups in each period was consistent, which did not affect the experimental results. However, this study has certain limitations, notably the imbalance in the gender distribution of participants, with a disproportionate number of males compared to females. This disparity could constrain the generalizability of the findings, particularly in relation to potential gender-specific pharmacokinetic differences. Blood samples were collected 22 times per period in the trial, and the results showed that AUC__%Extrap_ was less than 20% for all subjects, suggesting that the design of the blood collection time points was appropriate.

A 14-day washout period was employed to ensure the complete elimination of mifepristone during the first period. The elimination of mifepristone and its metabolites from the body is mainly through feces (83%) and urine (8.8%) within 6–7 days after the administration of a single oral dose (Sartor and Figg, 1996). According to the guiding principle of bioequivalence in the human body, the half-life of mifepristone in humans is 20–30 h (Heikinheimo et al., 2003). In our clinical trial, the elimination of the two preparations was similar, with the half-life of test preparation and reference preparation being, respectively, 19.80 ± 6.76 (34.14) hours and 19.00 ± 5.82 (30.65) hours. Thus, a 14-day washout period was selected to ensure complete metabolism of mifepristone.

Mifepristone, characterized by rapid absorption, is an orally active compound with a nearly 70% absorption rate, but its bioavailability is reduced to approximately 40% because of the first-pass effect, and the peak plasma concentrations are maintained at a relatively high level up to 48 or 72 h depending on the ingested dose (Heikinheimo et al., 2003; Sarkar, 2002). In the present study, following the oral administration of a single dose of 10 mg mifepristone, both the test and reference preparation of the peak took place approximately 0.75 h later, which was consistent with previously reported findings (Liao, Pang, Li, Xiong and Wu, 2008). The maximum plasma concentration of mifepristone in the present study was found to be 514.05 ± 177.65 (34.56) ng/mL and 502.68 ± 144.29 (28.70) ng/mL in test preparation and reference preparation, respectively, which were consistent with previously reported findings (Croxatto, Salvatierra, Croxatto and Fuentealba, 1993). However, peak plasma concentrations reached 0.36 ± 0.1, 1.2 ± 0.1, and 6.7 ± 3.4 mmol/L after the oral administration of 2, 8, and 25 mg mifepristone, respectively (Kekkonen, Heikinheimo, Mandelin and Lahteenmaki, 1996). In addition, in another report concerning mifepristone at single oral doses of 50, 75, and 100 mg in non-pregnant Chinese women, the peak plasma levels of mifepristone were 0.83 mcg/mL (1.91 μmol/L), 1.26 mcg/mL (2.90 μmol/L), and 1.65 mcg/mL (3.8 μmol/L), respectively (He et al., 1989; Liao et al., 2008; Sarkar, 2002; Shi et al., 1993). It seems that the pharmacokinetics of mifepristone is linear within the dose ranges of 50–100 mg/day and nonlinear within smaller doses. Different races may also lead to inconsistent results, which need to be further confirmed by more sample sizes. The concentration of mifepristone was below the lower limit of quantification in all subjects at the pre-dose sampling point. There is no statistically significant difference in C_max_ and AUC_0-t_ between the test preparation and reference preparation, indicating that the degree and rate of absorption for the two preparations were similar.

It is important to note that while specific studies investigating drug or food interactions with mifepristone are lacking, the drug is metabolized by the cytochrome P450 enzyme CYP3A4. Consequently, its metabolism may be inhibited by substances such as ketoconazole, itraconazole, erythromycin, and grapefruit juice, which could lead to increased serum levels of mifepristone. Conversely, agents such as rifampicin, dexamethasone, St. John’s wort, and certain anticonvulsants (including phenytoin, phenobarbital, and carbamazepine) may induce the metabolism of mifepristone, resulting in decreased serum concentrations of the drug. The metabolism of mifepristone generated by CYP3A4 to RU42633 and RU42698 was rapid and immunologically and biologically active and retained anti-progestational and anti-glucocorticoid properties just like mifepristone (Sartor and Figg, 1996). Therefore, the metabolites of mifepristone were also investigated in this study. The bioequivalence analysis showed that the 90% confidence intervals for the geometric mean ratios of PK parameters (C_max_, AUC_0-t_, and AUC_0-∞_) for the test and reference formulations and their primary metabolites (RU42633 and RU42698) fell within the bioequivalence range of 80.00%–125.00%. At the same time, there was no significant difference in T_max_ between test and reference preparations by the symbolic rank test. In addition, there were no serious adverse events in both groups. Elevations in serum enzyme and bilirubin levels have been documented in patients with Cushing syndrome undergoing long-term treatment with higher dosages of mifepristone (Funke and Rockey, 2019; Shah, Putnam, Daugherty, Vyas and Chuang, 2019). However, the mechanism of this elevation remains unclear. Hypotension has also been reported during post-approval long-term use of mifepristone with higher dosages (Wannachalee, Turcu and Auchus, 2018). Moreover, there was no significant difference in the incidence of adverse reactions between test and reference preparations. The results showed that reference preparation was bioequivalent to a single dose of mifepristone tablets and was safe for use in fasting Chinese healthy subjects.

## Data Availability

The original contributions presented in the study are included in the article/Supplementary Material; further inquiries can be directed to the corresponding authors.
